# Concomitant Ruxolitinib and Ibrutinib for Graft-Versus-Host Disease (GVHD): The First Reported Use in Pediatric Patients

**DOI:** 10.7759/cureus.29195

**Published:** 2022-09-15

**Authors:** Thomas A Gagliardi, Jordan Milner, Mitchell S Cairo, Amir Steinberg

**Affiliations:** 1 Hematology and Oncology, Westchester Medical Center, New York Medical College, Valhalla, USA; 2 Hematology and Oncology, Maria Fareri Children’s Hospital, New York Medical College, Valhalla, USA

**Keywords:** pediatrics, ruxolitinib, ibrutinib, clinical outcomes, hematopoietic stem cell transplantation, graft-versus-host disease

## Abstract

Allogeneic hematopoietic stem cell transplant (alloHSCT) can be a life-saving treatment for patients with hematological disorders but far too often carries the feared complication of graft-versus-host disease (GVHD). The first-line treatment of GVHD is typically corticosteroids, but steroid-refractory chronic GVHD (cGVHD) has led to the Food and Drug Administration (FDA) approval of ruxolitinib (Jakafi), ibrutinib (Imbruvica), and belumosudil (Rezurock).

Patient 1 was a four-year-old female diagnosed with natural killer (NK) cell dysfunction who underwent alloHSCT with cells from a 9/10 National Marrow Donor Program (NMDP) donor and subsequently developed chronic GVHD (cGVHD) of the skin and gut. This cGVHD was refractory to steroids and ibrutinib but improved with the administration of concomitant ibrutinib and ruxolitinib. Patient 2 was a one-year-old male with sickle cell anemia. The patient was transplanted under a haploidentical protocol from the mother but developed bronchiolitis obliterans organizing pneumonia (BOOP) and pathology-confirmed GVHD. This cGVHD was steroid-refractory and resolved with the administration of concomitant ibrutinib and ruxolitinib. To our knowledge, this is the first reported use of concomitant ruxolitinib and ibrutinib in pediatric patients. The combination was well tolerated with no significant adverse events. Neither patient had to discontinue these drugs. We propose a further investigation into this dual therapy in cGVHD either compared to steroids or as a second-line option.

## Introduction

Allogeneic hematopoietic stem cell transplant (alloHSCT) can be a life-saving treatment option for patients with hematological disorders. Unfortunately, 30%-70% of patients who undergo alloHSCT develop a complication known as graft-versus-host disease (GVHD) [1]. During GVHD, donor T and B lymphocytes become activated by antigen-presenting cells due to human leukocyte antigen (HLA) differences in recipient tissue. Subsequently, cellular and inflammatory factors damage target organs, especially the skin, gastrointestinal tract, lungs, and liver. In the past, tissue damage that occurred within 100 days of transplant had been deemed acute GVHD (aGVHD), while fibrosis affecting any organ after 100 days post-hematopoietic stem cell transplant (HSCT) had been called chronic GVHD (cGVHD) [2]. Today, it is the acuity of GVHD that determines whether it is deemed acute or chronic GVHD. The first-line treatment of both aGVHD and cGVHD is typically corticosteroids, which suppress the hyperactive immunological response. However, steroid-refractory GVHD has provided a challenge for patients and providers and led to the Food and Drug Administration (FDA) approval of ruxolitinib (Jakafi), ibrutinib (Imbruvica), and subsequently belumosudil (Rezurock), a selective Rho-associated coiled-coil containing protein kinase 2 (ROCK2) inhibitor, as the first therapies authorized for steroid-refractory cGVHD. Ibrutinib is an inhibitor of Bruton’s tyrosine kinase (BTK) and is approved to treat cGVHD as second-line therapy (≥1 prior therapy). Ruxolitinib is a Janus-associated kinase (JAK) inhibitor approved to treat aGVHD and was recently approved to treat cGVHD, also as second-line therapy. This approval was prompted by a successful phase III randomized trial that demonstrated the efficacy of ruxolitinib in treating glucocorticoid-refractory cGVHD when compared to the best available therapies, including ibrutinib [3]. However, this trial did not test ruxolitinib and ibrutinib in combination [3,4]. Both the JAK and BTK signaling pathways appear to be requisite for the development of cGVHD, making their therapeutic inhibition favorable [5,6]. Here, we describe two pediatric patients who received both drugs for their cGVHD.

This article was previously published as an abstract in the November 2021 supplemental issue of the journal Blood as part of the American Society of Hematology 2021 Conference [4].

## Case presentation

Case 1

Patient 1 was a four-year-old female who underwent alloHSCT due to a diagnosis of natural killer (NK) cell dysfunction. The relevant treatment modalities and diagnoses in a timeline in relation to alloHSCT during this patient’s care are depicted in Table 1 and are described in this paragraph. A conditioning regimen was implemented that consisted of the following and is depicted in Table 1. Alemtuzumab was given on day -11 at 2 mg/m^2^, on day -10 and -9 at 6 mg/m^2^, and on day -8 and -7 at 20 mg/m^2^. Fludarabine was given on days -7 through -3 at a dose of 30 mg/m^2^. Melphalan was given at a dose of 140 mg/m^2^ on day -2. The cells for the alloHSCT were collected from a 9/10 National Marrow Donor Program (NMDP) donor. The patient received a CD34+ enrichment with T cell addback of 2.1 × 10^5^ CD3/kg and was administered tacrolimus as GVHD prophylaxis [4]. On day +148, the patient developed aGVHD stage two, grade III (Glucksberg criteria) of the gut manifesting as diarrhea, which resolved following the administration of steroids, extracorporeal photopheresis (ECP), and mycophenolate mofetil (MMF). On day +189, the patient developed GVHD stage one, grade I of the skin that subsequently resolved [4]. About 15 months post-transplant, the patient began developing cGVHD of the skin and gut. This prompted the initiation of ibrutinib on day +490 at 140 mg daily. The cGVHD persisted despite ibrutinib, ECP, tacrolimus, and sirolimus. On day +883, ruxolitinib was initiated at 2.5 mg bis in die (BID) (twice per day), following which the patient demonstrated stable to slightly improved cGVHD. Ibrutinib was then tapered to 110 mg between days +951 and +980 [4]. The patient remained on ruxolitinib and ibrutinib as of day +1,172, and a four-week course of interleukin-2 (IL-2) was initiated on day +1,276 to elicit a better GVHD response (Table 1). No adverse events were reported for the combination of ruxolitinib and ibrutinib in this patient.

**Table 1:** Timeline of treatment modalities and diagnoses in relation to alloHSCT in patient 1. alloHSCT: allogeneic hematopoietic stem cell transplant; aGVHD: acute graft-versus-host disease; ECP: extracorporeal photopheresis; MMF: mycophenolate mofetil; GVHD: graft-versus-host disease; cGVHD: chronic graft-versus-host disease; BID: bis in die (twice per day); IL-2: interleukin-2

Day	-11	-10	-9	-8	-7	-3	-2	0	148	189	490	883	951	980	1,172	1,276
Treatment modality or diagnosis	Alemtuzumab 2 mg/m^2^	Alemtuzumab 6 mg/m^2^	Alemtuzumab 6 mg/m^2^	Alemtuzumab 20 mg/m^2^	Fludarabine 30 mg/m^2^		Melphalan 140 mg/m^2^	alloHSCT, CD34+ enrichment with T cell addback of 2.1 × 10^5^ CD3/kg	aGVHD of the gut - steroids, ECP, MMF	GVHD of the skin - self-resolved	cGVHD of the skin and gut - began ibrutinib at 140 mg daily	cGVHD persistence - began ruxolitinib at 2.5 mg BID	Ibrutinib taper to 110 mg begins	Ibrutinib taper to 110 mg completed	Ruxolitinib and ibrutinib continued	Four-week course of IL-2 initiated

Case 2

Patient 2 was a one-year-old male with sickle cell anemia who underwent alloHSCT utilizing a haploidentical protocol from the mother (source: bone marrow). The relevant treatment modalities and diagnoses in a timeline in relation to alloHSCT during this patient’s care are depicted in Table 2 and are described in this paragraph. A conditioning regimen was implemented and is depicted in Table 2. Fludarabine was given intravenously at a dose of 30 mg/m^2^ on days -15 through -11. Busulfan was given at a dose of 2 mg/kg intravenously on days -9 through -7. Thiotepa was given on day -6 at a dose of 10 mg/kg IV. Cyclophosphamide was given at a dose of 50 mg/kg IV with mesna on days -5 and -4. Thymoglobulin was given at a dose of 2 mg/kg on days -5 through -2 [4]. The patient received a CD34+ enrichment with T cell addback of 2 × 10^5^ CD3/kg. The patient began to exhibit constitutional symptoms including fever and dyspnea, and a computed tomography (CT) scan of the chest was performed that raised concern for an infiltrative process. These symptoms did not improve with the addition of broad-spectrum antibiotics. The patient was diagnosed with bronchiolitis obliterans organizing pneumonia (BOOP) on day +217 following a lung biopsy that was sent to pathology. After a review of the pathology report by an outside institution, the possibility of thrombotic microangiopathy (TMA) was raised in the context of high lactate dehydrogenase (LDH) and low platelets in the blood. To treat the BOOP, a regimen of fluticasone, azithromycin, and montelukast (FAM) was initiated [4]. Systemic steroids were not initiated at this time. Despite FAM, the BOOP persisted as confirmed by a lung biopsy on day +407 and was considered as cGVHD at this point. On day +411, the patient was placed on ibrutinib 140 mg daily and ruxolitinib 2.5 mg BID. On day +414, the patient was initiated on ECP twice per week. Symptoms began to improve after one month of this therapy. The state of the lungs appeared stable on computed tomography (CT) imaging following the initiation of ibrutinib, ruxolitinib, and ECP. Beginning on day +477, the dose of ruxolitinib was tapered in half [4]. The cGVHD and BOOP of the lungs have since resolved, and the patient was tapered off the three modalities sequentially. The patient was tapered off ruxolitinib by day +534, ibrutinib by day +618, and ECP by day +628 (Table 2). No adverse events were reported for the combination of ruxolitinib and ibrutinib in this patient.

**Table 2:** Timeline of treatment modalities and diagnoses in relation to alloHSCT in patient 2. alloHSCT: allogeneic hematopoietic stem cell transplant; BOOP: bronchiolitis obliterans organizing pneumonia; FAM: fluticasone, azithromycin, and montelukast; cGVHD: chronic graft-versus-host disease; BID: bis in die (twice per day); ECP: extracorporeal photopheresis

Day	-15	-11	-9	-7	-6	-5	-4	-2	0	217	407	411	414	477	534	618	628
Treatment modality or diagnosis	Fludarabine 30 mg/m^2^		Busulfan 2 mg/kg		Thiotepa 10 mg/kg	Cyclophosphamide 50 mg/kg, with mesna; thymoglobulin 2 mg/kg	Cyclophosphamide 50 mg/kg, with mesna; thymoglobulin 2 mg/kg	Thymoglobulin 2 mg/kg	alloHSCT, CD34+ enrichment with T cell addback of 2 × 10^5^ CD3/kg	Diagnosis of BOOP on pathology, FAM therapy	BOOP persistence confirmed by pathology - considered cGVHD	Ibrutinib 140 mg daily initiated; ruxolitinib 2.5 mg BID initiated	ECP twice per week initiated	Ruxolitinib tapered to half of the original dose due to clinical improvement	Ruxolitinib taper to 0 mg completed, ruxolitinib discontinued	Ibrutinib taper to 0 mg completed, ibrutinib discontinued	ECP discontinued

## Discussion

We believe this to be the first reported use of concomitant therapy of ruxolitinib and ibrutinib to treat cGVHD in the pediatric population. We identified one paper reporting on ruxolitinib for cGVHD that briefly mentioned three patients on ibrutinib simultaneously, but no further details were provided [7]. The cases presented here demonstrate the feasibility of administering ruxolitinib and ibrutinib concomitantly in the management of cGVHD. Our patients tolerated this dual-drug therapy well and did not experience any significant adverse events. Adjunct therapies such as ECP and IL-12 were also incorporated to improve the GVHD response. Ruxolitinib was recently approved by the Food and Drug Administration (FDA) as a therapy for cGVHD (September 2021), joining ibrutinib as tyrosine kinase inhibitors indicated for cGVHD. Ruxolitinib was approved for patients ages 12 and over, and ibrutinib was recently approved for patients ages 1-12 years at a dose of 240 mg/m^2^ daily and for patients 12 years and older at 420 mg/m^2^ daily. As a result of these approvals, we expect that this combined therapy will become more common going forward. Given our success with this experience, we have recently applied the concomitant ruxolitinib and ibrutinib approach to a third patient, and we hope for similar outcomes.

Chronic GVHD tends to persist and takes a long time to resolve. The manifestation of the GVHD and its prolonged duration can affect the quality of life of patients to a significant degree. By combining the agents ruxolitinib and ibrutinib, the hope is that not only will the response rate against chronic GVHD increase but also will the duration of involvement decrease by targeting GVHD from both the JAK and BTK pathways. This is why ruxolitinib was added to ibrutinib in patient 1 and both were started simultaneously in patient 2. It is possible that one drug alone may be adequate, but given the data using single agents, our team felt that dual therapy was an approach worth initiating in the two patients presented here. Simultaneously administering both agents may potentially have a favorable toxicity profile. There are individual agent toxicity concerns such as pancytopenia, fatigue, and gastrointestinal symptoms for ruxolitinib [8] and gastrointestinal toxicity, especially diarrhea, bleeding issues, high blood pressure, atrial fibrillation, and cytopenias for ibrutinib [9]. As one can see, these agents may share similar toxicities. By carefully monitoring symptoms and dose-adjusting, if needed, these side effects may be manageable. Additionally, our pediatric population may be better able to tolerate the adverse effects of combining both medications. We understand that only a randomized trial will truly be able to answer this important question of efficacy as well as safety. It would be meaningful to see data comparing these agents to steroids as front-line therapy for new chronic GVHD.

Our team was concerned with the toxicity of steroids and the concern for dependency on steroids in the pediatric population. Long-term steroid use can have debilitating effects on children, such as stunted growth, and effects on natural development. These issues are not relevant to the adult population. Children may also experience the same toxicities that adults can suffer with steroids, such as high blood pressure, bone strength and growth, hyperglycemia, and heart and liver toxicity, threatening long-term health. Although the novel immunosuppressive therapies ruxolitinib and ibrutinib are more expensive than steroids, one must keep in mind the financial costs of long-term steroid exposure, which can be significant over the course of one’s lifetime, especially in the pediatric population [10]. Thus, ruxolitinib or ibrutinib alone, or in combination as our group demonstrated, could be a potential treatment approach for newly diagnosed chronic GVHD in the pediatric population.

Further research should be done to better understand how the combined therapy of ruxolitinib and ibrutinib compares in the treatment of cGVHD to steroids. It is noteworthy that the FDA recently approved belumosudil (Rezurock) for cGVHD (July 2021). Belumosudil is a selective Rho-associated coiled-coil containing protein kinase 2 (ROCK2) inhibitor [11]. This decreases inflammation by decreasing the activation of signal transducer and activator of transcription 3 (STAT3), preventing the JAK2-STAT3 complex and thus downregulating Th17 and Tfh cells. The inhibition of ROCK2 also increases the phosphorylation of signal transducer and activator of transcription 5 (STAT5), upregulating Treg cells to decrease inflammation. Belumosudil also decreases fibrosis, which has been seen in animal models as decreased collagen deposition around the bronchioles and delayed progression of scleroderma [11]. This illustrates yet another pathway of inflammation and fibrosis that physicians can target in the therapy of GVHD.

The future of cGVHD management may benefit from “cocktails” of two, three, or even more drugs targeting distinct pathways to elicit a synergistic response against this common complication of bone marrow transplants. Since both the JAK and BTK signaling pathways appear requisite for the development of GVHD, it makes sense that targeting both at the same time could elicit a stronger response than selecting one at a time, hence our use of concomitant ruxolitinib and ibrutinib. However, the financial toxicity of these drugs is to be considered. These immunosuppressive medications can cause a substantial financial burden on patients and society, so the judicious use of such medication cocktails is recommended. On the horizon are additional therapies designed for cGVHD. Axatilimab, a humanized antibody against macrophage colony-stimulating factor receptor (CSF-1R), is in an active phase II clinical trial to dampen pulmonary fibrosis in cGVHD [12]. Abatacept (Orencia) is an immunomodulatory fusion protein currently being investigated for its efficacy in treating steroid-refractory cGVHD [13]. Abatacept recently became the first drug FDA approved specifically for aGVHD prophylaxis in December 2021 but is contraindicated in the presence of a JAK inhibitor, such as ruxolitinib. With the rapid development of these new therapies and the strategic combination of existing ones, the future of patients with cGVHD has solid grounds for hope.

## Conclusions

Pediatric patients suffering from steroid-refractory chronic graft-versus-host disease (cGVHD) may be effectively treated with concomitant ruxolitinib and ibrutinib without serious adverse effects related to the dual administration of these drugs. The suppression of both the JAK and BTK pathways simultaneously appears to reduce GVHD progression. Multidrug therapy can be considered to manage challenging cases of cGVHD while considering the financial burden that these drugs may have on patients and their families. Large-scale studies should be conducted to further evaluate the safety and efficacy of our proposed combination therapy. More therapies are likely to be approved for GVHD management, making it important for physicians to stay well-read on the changing landscape of GVHD treatment.
